# An Interesting Case of Serratia Endocarditis in a Patient With Chronic Myeloid Leukemia

**DOI:** 10.7759/cureus.21238

**Published:** 2022-01-14

**Authors:** Annapoorna Singh, Kathyayini Tappeta, Nikitha Chellapuram, Daulath Singh

**Affiliations:** 1 Internal Medicine, University of Missouri–Kansas City School of Medicine, Kansas City, USA; 2 Internal Medicine, St. George’s University School of Medicine, True Blue, GRD; 3 Internal Medicine, Mayo Clinic, Rochester, USA; 4 Internal Medicine/Hematology-Oncology, Stormont Vail Health, Topeka, USA

**Keywords:** infective endocarditis, immunocompromised patient, chronic myeloid leukemia (cml), serratia endocarditis, aortic endocarditis

## Abstract

*Serratia* is a rare cause of infective endocarditis (IE) and usually occurs in patients with underlying risk factors, such as intravenous (IV) drug use, and human immunodeficiency virus patients. Gram-negative bacteria endocarditis is associated with high mortality when it involves the left side of the heart and often requires surgical intervention in addition to medical treatment. Although most gram-negative endocarditis cases are hospital-acquired, community cases have also been reported. Here, we present a case of *Serratia *endocarditis in an individual who was later diagnosed with chronic myeloid leukemia (CML) during the same hospitalization. The patient was treated with IV meropenem and started on targeted therapy for CML. CML is presumed to have likely predisposed the patient to bacteremia and IE.

## Introduction

*Serratia* species are gram-negative bacilli of the Enterobacteriaceae group. The genus *Serratia* consists of at least 20 species and *S. marcescens* is the main human pathogen [1]. *S. marcescens* has been associated with urinary tract infections, pneumonia, and bloodstream infections [1]. Although endocarditis caused by *Serratia* species is rare, it has been previously reported in intravenous drug users (IVDUs), human immunodeficiency virus (HIV) patients, and patients with prosthetics heart valves; however, it has never been reported in patients with chronic myeloid leukemia (CML). Here, we report a case of *Serratia* native valve endocarditis in a patient who presented with multiple symptoms and was newly diagnosed with CML during the same hospitalization.

This case report was previously presented as a poster at the Midwest Clinical & Translational Research Meeting held on April 4-5, 2019 in Chicago, Illinois, USA.

## Case presentation

**Figure 1:**
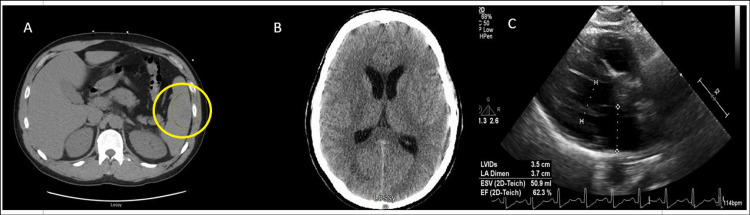
CT of the abdomen and pelvis showing mild hepatomegaly, mild splenomegaly, and possible splenic infarcts (A, yellow circle). CT of the head without contrast showing no acute intracranial process (B). TTE with a normal ejection fraction of 55%, no valvular vegetation, and BAV with mild stenosis (C). CT: computed tomography; TTE: transthoracic echocardiogram; BAV: bicuspid aortic valve

A 40-year-old Caucasian male with a history of bicuspid aortic valve (BAV) presented to our hospital with left upper quadrant abdominal pain, nausea, vomiting, diarrhea, urinary frequency, and lethargy for two days. The patient also had sudden onset of dizziness, rigors, and low back pain one day before the presentation. He visited an urgent care center where he was negative for influenza and was subsequently sent home. On presentation, body temperature was 103.1°F; laboratory values are listed in Table 1. Computed tomography (CT) of the abdomen and pelvis showed mild hepatomegaly, mild splenomegaly, and possible splenic infarcts (Figure 1A). CT of the head without contrast was performed due to acute encephalopathy which showed no acute intracranial process (Figure 1B). A transthoracic echocardiogram (TTE) showed a normal ejection fraction of 55%, no valvular vegetation, BAV with mild stenosis (mean gradient of 21 mmHg), and elevated central venous pressure (Figure 1C). Platelet counts were normal on presentation (Table 1) but dropped to 40,000/µL on the third day of hospitalization. Duplex ultrasound of lower extremities was negative for thrombosis. The hematology team performed a bone marrow biopsy due to leukocytosis to rule out the malignant process.

**Table 1:** Laboratory findings of the patient.

Labs	Lab values	Reference values
White blood cell count	79,000/µL	4.00–11.00/µL
Platelet count	170,000/µL	140,000–400,000/µL
Aspartate transaminase	78 IU/L	15–46 IU/L
Alanine transaminase	58 IU/L	0–49 IU/L
Total bilirubin	3.2 mg/dL	0.2–1.3 mg/dL
Serum creatinine	2.4 mg/dL	0.6–1.3 mg/dL
Urinalysis		
White blood cell count	11–20/HPF	0–3/HPF
Red blood cell count	6–10/HPF	0–3/HPF
Leukocyte esterase	Negative	Absent
Nitrites	Negative	Absent
Bacteria	Large	Absent
Lactate dehydrogenase	1,801 IU/L	313–618 IU/L
International normalized ratio	2.2	1
Partial thromboplastin time	24.5	11.4–15.0 seconds
Cerebrospinal fluid analysis		
White blood cell count	1,747/µL	0–5/µL
Neutrophils	91%	0–6%
Red blood cell count	<3,000/µL	0/µL
Glucose	39 mg/dL	40–70 mg/dL
Protein	102 mg/dl	15–60 mg/dL

**Figure 2:**
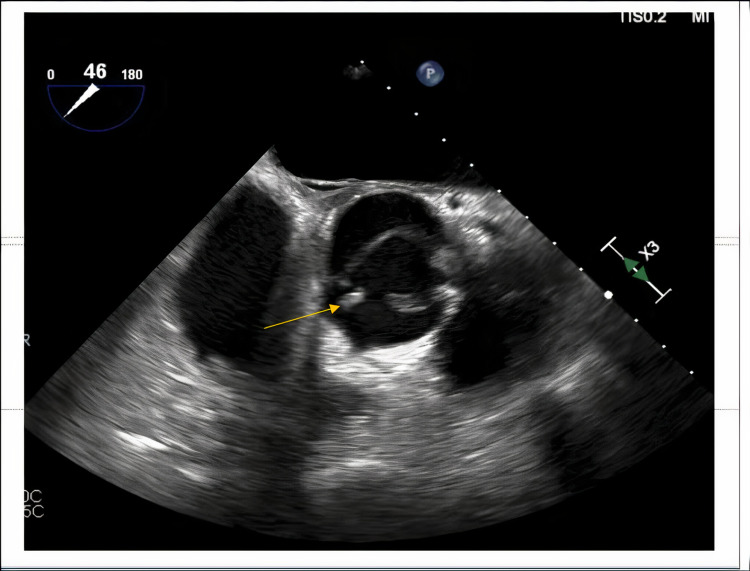
Transesophageal echocardiogram showing linear echo density measuring 1.9 cm on the aortic aspect of the aortic valve concerning for vegetation (yellow arrow).

**Figure 3:**
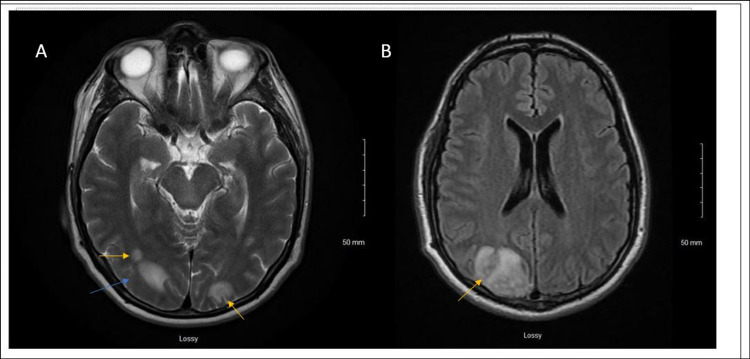
MRI of the brain with multiple brain abscesses secondary to septic emboli from aortic valve endocarditis (yellow arrows in A showing occipital emboli, blue arrow in A concerning for occipital abscess, yellow arrow in B showing a large occipital abscess). MRI: magnetic resonance imaging

The patient was empirically treated with IV ceftriaxone and vancomycin for possible sepsis. Urine culture grew *S. marcescens*. The patient continued to have fever, chills, and developed neck stiffness the following day of the admission; hence, a lumbar puncture was performed to rule out meningitis. Cerebrospinal fluid (CSF) analysis laboratory findings are listed in Table 1. Doxycycline was added to cover any tick-borne illnesses due to recent history of hiking. Infectious disease was consulted. Blood and CSF cultures were negative for any growth. CSF meningitis polymerase chain reaction panel was negative. A wide range of bacterial, viral, and fungal serologies, including tick and hepatitis panels, were performed all of which were negative. On day six of hospitalization, a transesophageal echocardiogram was performed as the patient continued to have high-grade fevers with chills and left upper quadrant abdominal pain. It showed linear echo density measuring 1.9 cm on the aortic aspect of the aortic valve concerning for vegetation (Figure 2). The intervalvular fibrosa appeared mildly thickened, although not diagnostic for abscess. MRI of the brain showed multiple brain abscesses secondary to septic emboli from aortic valve endocarditis (Figures 3A, 3B). Ceftriaxone was discontinued and meropenem was added to vancomycin and doxycycline. Anticoagulation was not initiated for splenic infarcts due to multiple brain abscesses.

**Figure 4:**
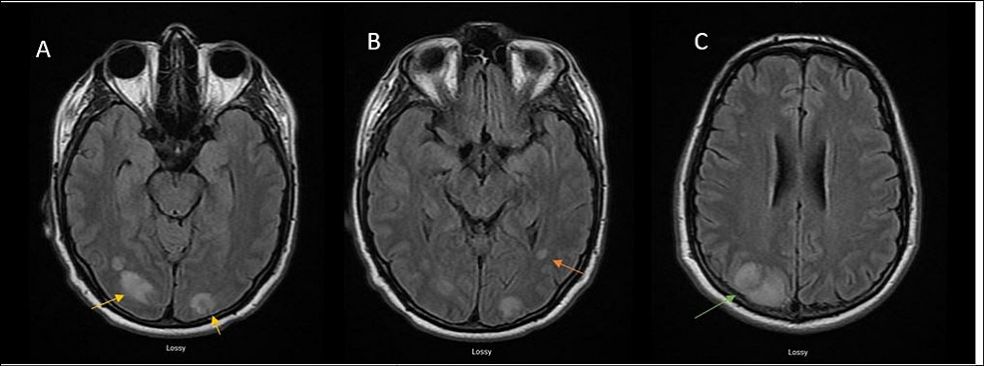
MRI of the brain showing multifocal lesions involving the bilateral occipital lobes (yellow arrow in A), parietal lobes (orange arrow in B), and cerebellum (green arrow in C) consistent with multifocal abscesses with edema. MRI: magnetic resonance imaging

Bone marrow biopsy showed hypercellular marrow with no increased blast population. Molecular testing was positive for BCR-ABL, consistent with the diagnosis of CML. MRI of the brain with/without contrast was repeated due to the worsening symptoms of occipital headache, new-onset blurring of vision, diplopia, nystagmus, vertigo, transient right facial droop, and left transient visual field defect. It showed acute new infarct in the right cerebellum, new abnormal flow within right vertebral artery suggestive of acute occlusion, demonstration of abnormal flow in the left vertebral artery suggestive of subacute or chronic occlusion, and multifocal lesions involving the cerebellum, bilateral occipital lobes, and parietal lobes consistent with multifocal abscesses with edema (Figures 4A-4C). The patient was then started on IV heparin for anticoagulation due to the risk of emboli propagation. At this time, vancomycin and doxycycline were discontinued and the patient was treated with meropenem and linezolid for endocarditis.

**Figure 5:**
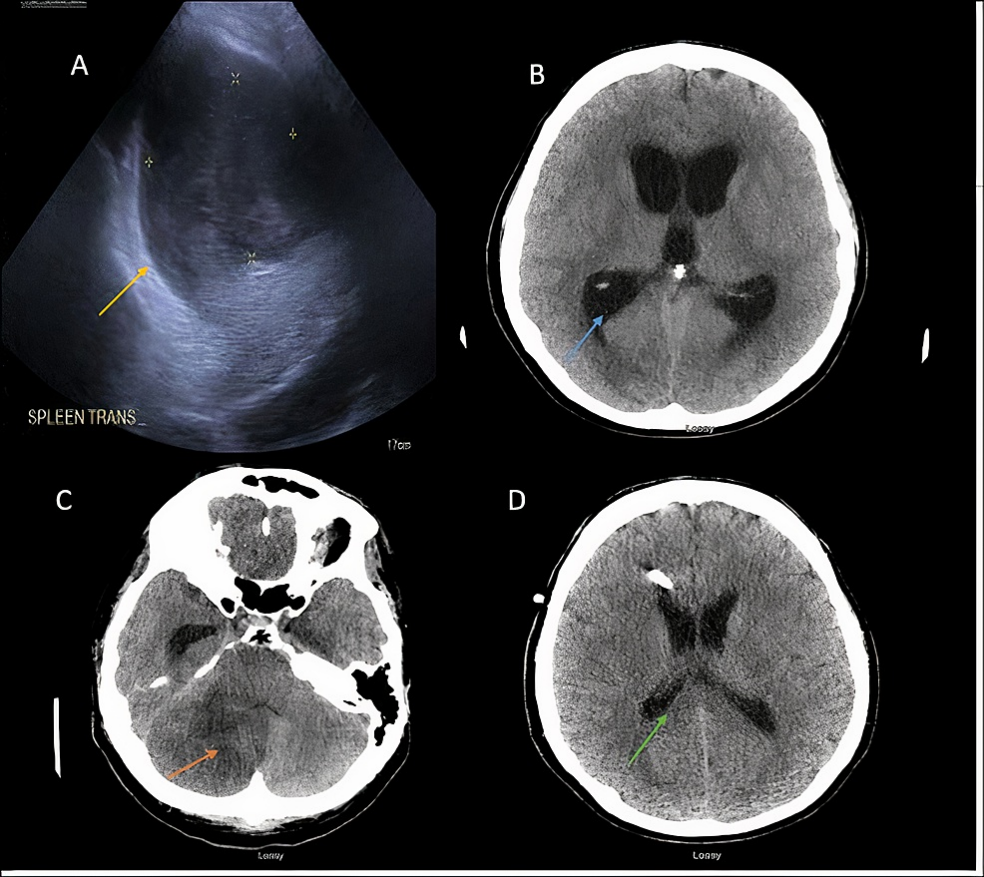
Ultrasound of the abdomen: large splenic abscess (yellow arrow in A). CT of the head without contrast showing acute hydrocephalus (blue arrow in B), and a large cerebellar infarct (orange arrow in C). Repeat CT of the head showing improvement in hydrocephalous (green arrow in D). CT: computed tomography

The patient had a repeat ultrasound of the abdomen that showed a large splenic abscess which prompted open splenectomy (Figure 5A). The decision was made to perform mechanical aortic valve replacement following the splenectomy for source control. Aortic valve vegetation was sent for microbiological analysis. Post-surgery, the patient developed confusion, worsening of the headache, and drowsiness. Repeat CT of the head without contrast revealed increased edema causing compression of the fourth ventricle resulting in acute hydrocephalus (Figure 5B), related to a large cerebellar infarct (Figure 5C). This was managed with hypertonic saline, acetazolamide, and the placement of an external ventricular drain. Approximately 10 days from admission, the patient was found to have progressively worsening thrombocytosis with platelets counts ranging 109,1000-184,1000/µL (reference range: 140,000-400,000/µL). The patient’s thrombocytosis was thought to be secondary to splenectomy; however, underlying infections/sepsis and CML could also have contributed. Hematology recommended hydroxyurea and the dose was gradually increased to 1 g twice daily. The patient’s neurological status gradually improved and a repeat CT scan of the head showed progressively decreasing fourth ventricle size (Figure 5D). The tube was taken out on the 25th day of admission. Heparin infusion was discontinued following the external ventricular drain removal and the patient was transitioned to warfarin for a prosthetic aortic valve. Meanwhile, splenic abscess culture grew *Candida albicans*, which was treated with fluconazole.

*S. marcescens* DNA was detected with the 16S rRNA gene primer set in the aortic vegetation after about three to four weeks of admission. The patient continued to have thrombocytosis, for which hydroxyurea dose was increased to 2 g daily and plateletpheresis was done to avoid thrombosis/bleeding complications. The patient was treated with a six-week course of meropenem for *Serratia* endocarditis with good response and was also initiated on treatment for CML prior to discharge.

## Discussion

*Serratia* species are facultatively anaerobic gram-negative bacillus of the Enterobacteriaceae group. *Serratia *species are motile and can adhere to cells via fimbriae. The genus *Serratia *consists of at least 20 species, of which eight are known to cause infections in humans [1]. *S. marcescens* is the main human pathogen and produces a few different hemolysins that are toxic to different cell types [2]. *S. marcescens* and several other *Serratia* species encode an inducible, chromosomal AmpC beta-lactamase, although it is typically expressed at low levels. The low levels at which this enzyme is typically expressed, AmpC beta-lactamase mediates resistance to several beta-lactam antibiotics, such as penicillin and first-generation cephalosporins [3].

Endocarditis is most commonly caused by gram-positive bacteria, and among the gram-negative organisms, *Escherichia* coli is the most common pathogen followed by *Pseudomonas aeruginosa* and *Klebsiella pneumoniae*, as reported in a prospective cohort study from 26 Italian centers. The genitourinary tract source, immunosuppression, and the presence of a cardiac implantable electronic device have been found to be associated with gram-negative bacillus endocarditis [4]. In another 12-year prospective cohort study of hospitalized patients from October 2002 to December 2014, among 284 patients, three bacterial species were isolated, namely, *Staphylococcus aureus*, *P. aeruginosa*, and *S. marcescens* [5]. Gram-negative bacillus bacteremia patients exhibit higher levels of comorbid conditions (hospital-acquired infection, malignancy, and recent surgery) [6]. In the same study, gram-negative bacillus endocarditis patients were more likely to have bacteremia originating from a gastrointestinal/genitourinary source which was probably the route of infection in our patient as the urine culture was positive for *Serratia*.

*Serratia *species has been occasionally recognized as the cause of healthcare-associated bacteremia due to the contamination of blood products, cleaning solutions, and inadequate sterile techniques with IV medications. However, a recent population-based study of *Serratia *species infections, including bacteremia in Canada, showed that 65% of infections were community-acquired; similar reports from Australia revealed that 47% of bacteremia episodes were community-acquired [7]. Although *S. marcescens* is the most common cause of endocarditis in the *Serratia *genus, cases of *S. liquefaciens* IE were reported in a patient with an intravascular central catheter (IVC)-related suppurative thrombophlebitis [8].

IE by *S. marcescens* was first described in the medical literature as a case series of 19 patients observed in the San Francisco Bay Area, 12 of whom were IVDU [9]. Endocarditis caused by *S. marcescens* has been seen most commonly in IVDU, and in one report it caused 14% of all addict-associated endocarditis cases [9]. Among these patients, most cases of right-sided endocarditis were cured by antibiotics alone while most cases of left-sided endocarditis treated medically alone did not survive. Most other cases of endocarditis caused by *S. marcescens* have occurred in patients with prosthetic heart valves. Only two cases of native valve endocarditis due to *S. marcescens* have been reported in the literature [10,11]. Both had right-sided involvement and indwelling IVC at the time of diagnosis and were successfully treated medically. An experimental endocarditis rabbit model using *S. marcescens* confirmed the significance of the presence of an indwelling catheter in the development of endocarditis [12].

In IVDU, injection of foreign antigens can precipitate antibody production that leads to immune complex deposition on valvular surfaces, which forms nidi for bacterial adhesion [13]. Studies have shown that IVDUs have “immunologic dysregulation” even when not HIV infected. It has been noted that HIV seropositive patients with CD4 cell counts of >350 cells/µL had an odds ratio (OR) of 2.31 for developing IE, whereas those with a CD4 cell count of <350 cells/µL had an OR of 8.31 [14]. Patients with abnormal immune functioning may not be unable to clear bacteria and are at risk of severe sepsis. The prognosis of endocarditis is also influenced by the immune status of the patient [15]. The evolution of vegetation size, its mobility and consistency, the extent of the disease, and the severity of valvular regurgitation have been related to late complications. With therapeutic options including modern antibiotic treatment and early surgical intervention, IE turned out to be a curable disease [16]. A case of ciprofloxacin-resistant *S. marcescens* in a non-Hodgkin lymphoma who was receiving ciprofloxacin for neutropenic prophylaxis has been reported but there are no case reports involving CML patients [17], as in our patient.

*Serratia *endocarditis is left-sided in IVDU [18]. Forty percent of *S. marcescens* endocarditis cases involve AV and septic embolization is common [19], which is consistent with our case. Because mortality rates are high with just medical therapy [19], a combination of medical and surgical management is recommended for left-sided endocarditis by *Serratia*. Most cases of right-sided endocarditis resolve with appropriate antibiotic administration without the need for further surgical management [20].

## Conclusions

There are several unique features in our reported case. As mentioned, our patient had no commonly reported risk factors for *Serratia *endocarditis such as IVDU, cardiac devices, prosthetic heart valve, and indwelling catheters. He had left-sided endocarditis with involvement of the native aortic valve which was not initially detected on the TTE. Because mortality rates are high with just medical therapy, a combination of medical and surgical management is recommended for left-sided endocarditis; most cases of right-sided endocarditis resolve with medical management. Our patient needed surgical intervention due to progressive embolic phenomena despite the medical therapy and size of the vegetation (1.9 cm). Another unique feature is that the patient survived after a morbid hospital course and multiple surgical procedures. He had underlying CML which might have led to an immunocompromised state and subsequent superinfection with *Serratia *from the urinary source and its subsequent dissemination. To our knowledge, this is the first reported case in the literature of a patient with left-sided *Serratia *endocarditis and underlying CML.
